# Supplementary material for: Reducing antibiotic prescribing in primary care in England from 2014 to 2017: population-based cohort study
Source: BMJ Open. 2019 Jul 9;9(7):e023989. doi: 10.1136/bmjopen-2018-023989 (PMC6661619; doi:10.1136/bmjopen-2018-023989)
Supplement: Supplementary data [file bmjopen-2018-023989supp001.pdf]

SUPPLEMENTARY DATA

Xiaohui Sun: Reducing antibiotic prescribing in primary care in England from 2014 to 2017. Population-based cohort study

Supplementary Table 1: Numbers of general practices contributing to CPRD from 2014 to 2017. Figures are mid-year counts.

**Variable**	**2014**	**2015**	**2016**	**2017**
All CPRD general practices	491	422	338	285
CPRD general practices in England	329	260	180	133
CPRD general practices in England participating in data linkage	257	202	138	102

Supplementary Table 2: Read codes for respiratory illness.

readcode	readterm
1656	Feverish cold
1712	Dry cough
1713	Productive cough -clear sputum
1714	Productive cough -green sputum
1715	Productive cough-yellow sputum
1716	Productive cough NOS
1716.11	Coughing up phlegm
1717	Night cough present
1719	Chesty cough
1719.11	Bronchial cough
1739	Shortness of breath
14B2.00	H/O: pneumonia
14B3.11	H/O: bronchitis
14B9.00	History of acute lower respiratory tract infection
16L..00	Influenza-like symptoms
171..00	Cough
171..11	C/O - cough
171A.00	Chronic cough
171B.00	Persistent cough
171C.00	Morning cough
171D.00	Evening cough
171E.00	Unexplained cough
171F.00	Cough with fever
171G.00	Bovine cough
171H.00	Difficulty in coughing up sputum
171J.00	Reflux cough
171K.00	Barking cough
171Z.00	Cough symptom NOS
173..00	Breathlessness
173B.00	Nocturnal cough / wheeze
1BA5.11	Pain in sinuses
1c3..00	Earache symptoms
1C3..00	Earache symptoms
1C32.00	Unilateral earache
1C33.00	Bilateral earache
1C3Z.00	Earache symptom NOS
1C9..00	Sore throat symptom
1C9..11	Throat soreness
1C92.00	Has a sore throat
1C93.00	Persistent sore throat
1C9Z.00	Sore throat symptom NOS

1CB..00	Throat symptom NOS
1CB3.00	Throat pain
1CB3.11	Pain in throat
1CB4.00	Feeling of lump in throat
1CB4.11	Constriction in throat
1CB4.12	Tightness in throat
1CB5.00	Throat irritation
1CBZ.00	Throat symptom NOS
2DB6.00	O/E - follicular tonsillitis
2DC2.00	O/E - granular pharyngitis
2DC3.00	Inflamed throat
A022200	Salmonella pneumonia
A32..00	Diphtheria
A320.00	Faucial diphtheria
A321.00	Nasopharyngeal diphtheria
A322.00	Anterior nasal diphtheria
A323.00	Laryngeal diphtheria
A32y.00	Other specified diphtheria
A32y000	Conjunctival diphtheria
A32y400	Cutaneous diphtheria
A32yz00	Other specified diphtheria NOS
A32z.00	Diphtheria NOS
A33..00	Whooping cough
A33..11	Bordetella
A330.00	Bordetella pertussis
A331.00	Bordetella parapertussis
A33y.00	Whooping cough - other specified organism
A33y000	Bordetella bronchiseptica
A33yz00	Other whooping cough NOS
A33z.00	Whooping cough NOS
A34..00	Streptococcal sore throat and scarlatina
A340.00	Streptococcal sore throat
A340100	Streptococcal laryngitis
A340200	Streptococcal pharyngitis
A340300	Streptococcal tonsillitis
A340z00	Streptococcal sore throat NOS
A34z.00	Streptococcal sore throat with scarlatina NOS
A383000	Fusobacterial necrotising tonsillitis
A54x400	Herpes simplex pneumonia
A551.00	Postmeasles pneumonia
A552.00	Postmeasles otitis media
A730.00	Ornithosis with pneumonia
A789300	HIV disease resulting in Pneumocystis carinii pneumonia
A789311	HIV disease resulting in Pneumocystis jirovecii pneumonia
AA12.00	Vincent's pharyngitis

AA1z.11	Vincent's laryngitis
AA1z.12	Vincent's tonsillitis
AA25.11	Rhinopharyngitis mutilans
AB24.11	Pneumonia - candidal
AB40500	Histoplasma capsulatum with pneumonia
AB41500	Histoplasma duboisii with pneumonia
AyuK900	[X]Mycoplasma pneumoniae [PPLO]cause/dis classifd/oth chaptr
AyuKA00	[X]Klebsiella pneumoniae/cause/disease classifd/oth chapters
F00y400	Meningitis due to klebsiella pneumoniae
F501.00	Infective otitis externa
F51..00	Nonsuppurative otitis media + eustachian tube disorders
F510.00	Acute non suppurative otitis media
F510000	Acute otitis media with effusion
F510011	Acute secretory otitis media
F510100	Acute serous otitis media
F510200	Acute mucoid otitis media
F510300	Acute sanguinous otitis media
F510z00	Acute nonsuppurative otitis media NOS
F514.00	Unspecified nonsuppurative otitis media
F514100	Serous otitis media NOS
F514200	Catarrhal otitis media NOS
F514300	Mucoid otitis media NOS
F514z00	Nonsuppurative otitis media NOS
F52..00	Suppurative and unspecified otitis media
F520.00	Acute suppurative otitis media
F520000	Acute suppurative otitis media tympanic membrane intact
F520100	Acute suppurative otitis media tympanic membrane ruptured
F520300	Acute suppurative otitis media due to disease EC
F520z00	Acute suppurative otitis media NOS
F521.00	Chronic suppurative otitis media, tubotympanic
F522.00	Chronic suppurative otitis media, atticoantral
F523.00	Chronic suppurative otitis media NOS
F524.00	Purulent otitis media NOS
F524000	Bilateral suppurative otitis media
F525.00	Recurrent acute otitis media
F526.00	Acute left otitis media
F527.00	Acute right otitis media
F528.00	Acute bilateral otitis media
F52z.00	Otitis media NOS
F52z.11	Infection ear
F53..00	Mastoiditis and related conditions
F530.00	Acute mastoiditis
F530.11	Abscess of mastoid

F530.12	Empyema of mastoid
F530000	Acute mastoiditis without complications
F530100	Subperiosteal mastoid abscess
F530200	Gradenigo's syndrome
F530300	Acute mastoiditis with other complication
F530z00	Acute mastoiditis NOS
F531.00	Chronic mastoiditis
F531000	Caries of mastoid
F531100	Post aural mastoid fistula
F531z00	Chronic mastoiditis NOS
F532.00	Petrositis
F5329	Petrositis
F533.00	Postmastoidectomy complication
F533000	Unspecified postmastoidectomy complication
F533100	Postmastoidectomy cavity mucinous cyst
F533200	Recurrent cholesteatoma postmastoidectomy
F533300	Postmastoidectomy granulation cavity
F533z00	Postmastoidectomy complication NOS
F53y.00	Other mastoid disorders
F53y000	Postauricular fistula
F53y100	Other mastoid disorder NOS
F53z.00	Mastoiditis NOS
F540.00	Acute myringitis without otitis media
F540z00	Acute myringitis NOS
F587.00	Otalgia
F587.11	Ear pain
FyuP000	[X]Other acute nonsuppurative otitis media
FyuP200	[X]Other chronic suppurative otitis media
FyuP300	[X]Otitis media in bacterial diseases classified elsewhere
FyuP400	[X]Otitis media in viral diseases classified elsewhere
FyuP500	[X]Otitis media in other diseases classified elsewhere
H0...00	Acute respiratory infections
H00..00	Acute nasopharyngitis
H00..11	Common cold
H00..12	Coryza - acute
H00..13	Febrile cold
H00..14	Nasal catarrh - acute
H00..15	Pyrexial cold
H00..16	Rhinitis - acute
H01..00	Acute sinusitis
H01..11	Sinusitis
H010.00	Acute maxillary sinusitis
H010.11	Antritis - acute
H011.00	Acute frontal sinusitis
H012.00	Acute ethmoidal sinusitis

H013.00	Acute sphenoidal sinusitis
H014.00	Acute rhinosinusitis
H01y.00	Other acute sinusitis
H01y000	Acute pansinusitis
H01yz00	Other acute sinusitis NOS
H01z.00	Acute sinusitis NOS
H02..00	Acute pharyngitis
H02..11	Sore throat NOS
H02..12	Viral sore throat NOS
H02..13	Throat infection - pharyngitis
H020.00	Acute gangrenous pharyngitis
H021.00	Acute phlegmonous pharyngitis
H022.00	Acute ulcerative pharyngitis
H023.00	Acute bacterial pharyngitis
H023000	Acute pneumococcal pharyngitis
H023100	Acute staphylococcal pharyngitis
H023z00	Acute bacterial pharyngitis NOS
H024.00	Acute viral pharyngitis
H02z.00	Acute pharyngitis NOS
H03..00	Acute tonsillitis
H03..11	Throat infection - tonsillitis
H03..12	Tonsillitis
H030.00	Acute erythematous tonsillitis
H031.00	Acute follicular tonsillitis
H032.00	Acute ulcerative tonsillitis
H033.00	Acute catarrhal tonsillitis
H034.00	Acute gangrenous tonsillitis
H035.00	Acute bacterial tonsillitis
H035000	Acute pneumococcal tonsillitis
H035100	Acute staphylococcal tonsillitis
H035z00	Acute bacterial tonsillitis NOS
H036.00	Acute viral tonsillitis
H037.00	Recurrent acute tonsillitis
H03z.00	Acute tonsillitis NOS
H04..00	Acute laryngitis and tracheitis
H040.00	Acute laryngitis
H040000	Acute oedematous laryngitis
H040100	Acute ulcerative laryngitis
H040200	Acute catarrhal laryngitis
H040300	Acute phlegmonous laryngitis
H040400	Acute haemophilus influenzae laryngitis
H040600	Acute suppurative laryngitis
H040w00	Acute viral laryngitis unspecified
H040x00	Acute bacterial laryngitis unspecified
H040z00	Acute laryngitis NOS
H041.00	Acute tracheitis

H041000	Acute tracheitis without obstruction
H041100	Acute tracheitis with obstruction
H041z00	Acute tracheitis NOS
H042.00	Acute laryngotracheitis
H042.11	Laryngotracheitis
H042000	Acute laryngotracheitis without obstruction
H042100	Acute laryngotracheitis with obstruction
H042z00	Acute laryngotracheitis NOS
H043.00	Acute epiglottitis (non strep)
H043.11	Viral epiglottitis
H043000	Acute epiglottitis without obstruction
H043100	Acute epiglottitis with obstruction
H043200	Acute obstructive laryngitis
H043211	Croup
H043z00	Acute epiglottitis NOS
H044.00	Croup
H04z.00	Acute laryngitis and tracheitis NOS
H05..00	Other acute upper respiratory infections
H050.00	Acute laryngopharyngitis
H051.00	Acute upper respiratory tract infection
H052.00	Pharyngotracheitis
H053.00	Tracheopharyngitis
H054.00	Recurrent upper respiratory tract infection
H055.00	Pharyngolaryngitis
H05y.00	Other upper respiratory infections of multiple sites
H05z.00	Upper respiratory infection NOS
H05z.11	Upper respiratory tract infection NOS
H05z.12	Viral upper respiratory tract infection NOS
H06..00	Acute bronchitis and bronchiolitis
H060.00	Acute bronchitis
H060.11	Acute wheezy bronchitis
H060000	Acute fibrinous bronchitis
H060100	Acute membranous bronchitis
H060200	Acute pseudomembranous bronchitis
H060300	Acute purulent bronchitis
H060400	Acute croupous bronchitis
H060500	Acute tracheobronchitis
H060600	Acute pneumococcal bronchitis
H060700	Acute streptococcal bronchitis
H060800	Acute haemophilus influenzae bronchitis
H060900	Acute neisseria catarrhalis bronchitis
H060A00	Acute bronchitis due to mycoplasma pneumoniae
H060B00	Acute bronchitis due to coxsackievirus
H060C00	Acute bronchitis due to parainfluenza virus
H060D00	Acute bronchitis due to respiratory syncytial virus
H060E00	Acute bronchitis due to rhinovirus

H060F00	Acute bronchitis due to echovirus
H060v00	Subacute bronchitis unspecified
H060w00	Acute viral bronchitis unspecified
H060x00	Acute bacterial bronchitis unspecified
H060z00	Acute bronchitis NOS
H061.00	Acute bronchiolitis
H061000	Acute capillary bronchiolitis
H061100	Acute obliterating bronchiolitis
H061200	Acute bronchiolitis with bronchospasm
H061300	Acute exudative bronchiolitis
H061500	Acute bronchiolitis due to respiratory syncytial virus
H061600	Acute bronchiolitis due to other specified organisms
H061z00	Acute bronchiolitis NOS
H062.00	Acute lower respiratory tract infection
H06z.00	Acute bronchitis or bronchiolitis NOS
H06z000	Chest infection NOS
H06z011	Chest infection
H06z100	Lower resp tract infection
H06z111	Respiratory tract infection
H06z112	Acute lower respiratory tract infection
H06z200	Recurrent chest infection
H07..00	Chest cold
H0y..00	Other specified acute respiratory infections
H0z..00	Acute respiratory infection NOS
H121100	Atrophic pharyngitis
H121200	Granular pharyngitis
H121300	Hypertrophic pharyngitis
H121400	Pharyngitis keratosa
H130.12	Maxillary sinusitis
H131.11	Frontal sinusitis
H135.00	Recurrent sinusitis
H13y100	Pansinusitis
H14y600	Lingular tonsillitis
H2...00	Pneumonia and influenza
H20..00	Viral pneumonia
H20..11	Chest infection - viral pneumonia
H200.00	Pneumonia due to adenovirus
H201.00	Pneumonia due to respiratory syncytial virus
H202.00	Pneumonia due to parainfluenza virus
H203.00	Pneumonia due to human metapneumovirus
H20y.00	Viral pneumonia NEC
H20y000	Severe acute respiratory syndrome
H20z.00	Viral pneumonia NOS
H21..00	Lobar (pneumococcal) pneumonia
H21..11	Chest infection - pneumococcal pneumonia
H22..00	Other bacterial pneumonia

H22..11	Chest infection - other bacterial pneumonia
H220.00	Pneumonia due to klebsiella pneumoniae
H221.00	Pneumonia due to pseudomonas
H222.00	Pneumonia due to haemophilus influenzae
H222.11	Pneumonia due to haemophilus influenzae
H223.00	Pneumonia due to streptococcus
H223000	Pneumonia due to streptococcus, group B
H224.00	Pneumonia due to staphylococcus
H22y.00	Pneumonia due to other specified bacteria
H22y000	Pneumonia due to escherichia coli
H22y011	E.coli pneumonia
H22y100	Pneumonia due to proteus
H22y200	Pneumonia - Legionella
H22yX00	Pneumonia due to other aerobic gram-negative bacteria
H22yz00	Pneumonia due to bacteria NOS
H22z.00	Bacterial pneumonia NOS
H23..00	Pneumonia due to other specified organisms
H23..11	Chest infection - pneumonia organism OS
H230.00	Pneumonia due to Eaton's agent
H231.00	Pneumonia due to mycoplasma pneumoniae
H232.00	Pneumonia due to pleuropneumonia like organisms
H233.00	Chlamydial pneumonia
H23z.00	Pneumonia due to specified organism NOS
H24..00	Pneumonia with infectious diseases EC
H24..11	Chest infection with infectious disease EC
H240.00	Pneumonia with measles
H241.00	Pneumonia with cytomegalic inclusion disease
H242.00	Pneumonia with ornithosis
H243.00	Pneumonia with whooping cough
H243.11	Pneumonia with pertussis
H244.00	Pneumonia with tularaemia
H246.00	Pneumonia with aspergillosis
H247000	Pneumonia with candidiasis
H247100	Pneumonia with coccidioidomycosis
H247z00	Pneumonia with systemic mycosis NOS
H24y.00	Pneumonia with other infectious diseases EC
H24y000	Pneumonia with actinomycosis
H24y100	Pneumonia with nocardiasis
H24y200	Pneumonia with pneumocystis carinii
H24y300	Pneumonia with Q-fever
H24y400	Pneumonia with salmonellosis
H24y500	Pneumonia with toxoplasmosis
H24y600	Pneumonia with typhoid fever
H24y700	Pneumonia with varicella
H24yz00	Pneumonia with other infectious diseases EC NOS

H24z.00	Pneumonia with infectious diseases EC NOS
H25..00	Bronchopneumonia due to unspecified organism
H25..11	Chest infection - unspecified bronchopneumonia
H26..00	Pneumonia due to unspecified organism
H26..11	Chest infection - pnemonia due to unspecified organism
H260.00	Lobar pneumonia due to unspecified organism
H260000	Lung consolidation
H261.00	Basal pneumonia due to unspecified organism
H262.00	Postoperative pneumonia
H263.00	Pneumonitis, unspecified
H27..00	Influenza
H270.00	Influenza with pneumonia
H270.11	Chest infection - influenza with pneumonia
H270000	Influenza with bronchopneumonia
H270100	Influenza with pneumonia, influenza virus identified
H270z00	Influenza with pneumonia NOS
H271.00	Influenza with other respiratory manifestation
H271000	Influenza with laryngitis
H271100	Influenza with pharyngitis
H271z00	Influenza with respiratory manifestations NOS
H27y.00	Influenza with other manifestations
H27y100	Influenza with gastrointestinal tract involvement
H27yz00	Influenza with other manifestations NOS
H27z.00	Influenza NOS
H27z.11	Flu like illness
H27z.12	Influenza like illness
H28..00	Atypical pneumonia
H29..00	Avian influenza
H2A..00	Influenza due to Influenza A virus subtype H1N1
H2A..11	Influenza A (H1N1) swine flu
H2B..00	Community acquired pneumonia
H2C..00	Hospital acquired pneumonia
H2y..00	Other specified pneumonia or influenza
H2z..00	Pneumonia or influenza NOS
H30..00	Bronchitis unspecified
H30..11	Chest infection - unspecified bronchitis
H300.00	Tracheobronchitis NOS
H301.00	Laryngotracheobronchitis
H30z.00	Bronchitis NOS
H310100	Smokers' cough
H312200	Acute exacerbation of chronic obstructive airways disease
H470.11	Aspiration pneumonia
H470312	Aspiration pneumonia due to vomit
H471000	Lipoid pneumonia (exogenous)
H50..00	Empyema

H500.00	Empyema with fistula
H500000	Empyema with bronchocutaneous fistula
H500100	Empyema with bronchopleural fistula
H500400	Empyema with pleural fistula NOS
H501.00	Empyema with no fistula
H501000	Pleural abscess
H501100	Thorax abscess NOS
H501200	Pleural empyema
H501300	Lung empyema NOS
H501400	Purulent pleurisy
H501500	Pyopneumothorax
H501600	Pyothorax
H50z.00	Empyema NOS
H51..00	Pleurisy
H510.00	Pleurisy without effusion or active tuberculosis
H510000	Adhesion of pleura or lung
H510100	Thickening of pleura
H510200	Calcification of pleura
H510300	Acute dry pleurisy
H510400	Diaphragmatic pleurisy
H510500	Basal pleurisy
H510600	Chronic dry pleurisy
H510700	Fibrinous pleurisy
H510800	Sterile pleurisy
H510900	Pneumococcal pleurisy
H510A00	Staphylococcal pleurisy
H510B00	Streptococcal pleurisy
H510C00	Pleural plaque
H510z00	Pleurisy without effusion or active tuberculosis NOS
H511.00	Bacterial pleurisy with effusion
H511000	Pneumococcal pleurisy with effusion
H511100	Staphylococcal pleurisy with effusion
H511200	Streptococcal pleurisy with effusion
H511z00	Bacterial pleurisy with effusion NOS
H51y.00	Other pleural effusion excluding mention of tuberculosis
H51y000	Encysted pleurisy
H51y100	Haemopneumothorax
H51y200	Haemothorax
H51y300	Hydropneumothorax
H51y400	Hydrothorax
H51y500	Chylous effusion
H51y600	Fibrothorax
H51y700	Malignant pleural effusion
H51yz00	Other pleural effusion
H51z.00	Pleural effusion NOS

H51z000	Exudative pleurisy NOS
H51z100	Serofibrinous pleurisy NOS
H51z200	Serous pleurisy NOS
H51zz00	Pleural effusion NOS
H530200	Gangrenous pneumonia
H530300	Abscess of lung with pneumonia
H540000	Hypostatic pneumonia
H540100	Hypostatic bronchopneumonia
Hyu0.00	[X]Acute upper respiratory infections
Hyu0000	[X]Other acute sinusitis
Hyu0100	[X]Acute pharyngitis due to other specified organisms
Hyu0200	[X]Acute tonsillitis due to other specified organisms
Hyu0300	[X]Other acute upper respiratory infections/multiple sites
Hyu0500	[X]Influenza+other manifestations,influenza virus identified
Hyu0600	[X]Influenza+oth respiratory manifestatns,virus not identifd
Hyu0700	[X]Influenza+other manifestations, virus not identified
Hyu0800	[X]Other viral pneumonia
Hyu0A00	[X]Other bacterial pneumonia
Hyu0B00	[X]Pneumonia due to other specified infectious organisms
Hyu0D00	[X]Pneumonia in viral diseases classified elsewhere
Hyu0H00	[X]Other pneumonia, organism unspecified
Hyu1.00	[X]Other acute lower respiratory infections
Hyu1000	[X]Acute bronchitis due to other specified organisms
Hyu1100	[X]Acute bronchiolitis due to other specified organisms
M03z000	Cellulitis NOS
R041.00	[D]Throat pain
R062.00	[D]Cough
SN30.11	Aero-otitis media
SN31.11	Aerosinusitis
SP13100	Other aspiration pneumonia as a complication of care
SP13200	Post operative chest infection

Supplementary Table 3: Read codes for genitourinary conditions.

readcode	readterm
1979	Suprapubic pain
14D2.00	H/O: kidney infection
14D4.00	H/O: recurrent cystitis
14D5.00	H/O: haematuria
14D6.00	H/O: urethral stricture
14D7.00	History of recurrent urinary tract infection
14DZ.00	H/O: urinary disease NOS
16F..00	Double incontinence
1A...00	Genitourinary symptoms
1A...11	GU symptoms
1A...12	Urinary symptoms
1A1..00	Micturition frequency
1A1..11	Frequency of micturition
1A1..12	Polyuria
1A1..13	Urinary frequency
1A12.00	Frequency of micturition
1A13.00	Nocturia
1A1Z.00	Micturition frequency NOS
1A2..00	Micturition control
1A2..11	Urinary control
1A22.00	Enuresis
1A22000	Nocturnal enuresis
1A22011	Bedwetting
1A22100	Daytime enuresis
1A23.00	Incontinence of urine
1A24.00	Stress incontinence
1A24.11	Stress incontinence - symptom
1A25.00	Urgency
1A25.11	Urgency of micturition
1A26.00	Urge incontinence of urine
1A27.00	Urge to pass urine again shortly after finishing voiding
1A2Z.00	Micturition control NOS
1A3..00	Micturition stream
1A3..11	Urine stream
1A32.00	Cannot pass urine - retention
1A32.11	Retention - symptom
1A33.00	Micturition stream poor
1A34.00	Hesitancy
1A34.11	Hesitancy of micturition
1A35.00	Precipitancy
1A35.11	Precipitancy of micturition
1A36.00	Terminal dribbling of urine

1A37.00	Dribbling of urine
1A3Z.00	Micturition stream NOS
1A4..00	Urine appearance
1A4..11	Urine appearance symptom
1A41.00	Urine looks normal
1A42.00	Urine looks dark
1A43.00	Urine looks pale
1A44.00	Urine looks cloudy
1A45.00	Blood in urine - haematuria
1A45.11	Blood in urine - symptom
1A45.12	Haematuria - symptom
1A4Z.00	Urine appearance NOS
1A5..00	Genitourinary pain
1A51.00	No genitourinary pain
1A52.00	Renal colic
1A52.11	Renal colic, symptom
1A53.00	Lumbar ache - renal
1A53.11	C/O - loin pain
1A53.12	C/O - lumbar pain
1A53.13	C/O - renal pain
1A54.00	Ureteric colic
1A54.11	C/O - ureteric colic
1A54.12	C/O - ureteric pain
1A55.00	Dysuria
1A56.00	Strangury
1A57.00	Pain in testicle
1A57.11	Testicular pain
1A5B.00	Pain in penis
1A5D.00	Urethral pain
1A5Z.00	Genitourinary pain NOS
1A6..00	Urethral discharge symptom
1A61.00	No urethral discharge
1A62.00	Urethral discharge
1A6Z.00	Urethral discharge NOS
1AC..00	Micturition volume
1AC0.00	Anuria
1AC1.00	Oligouria
1AC2.00	Polyuria
1AF..00	Diuresis
1AG..00	Recurrent urinary tract infections
1AH..00	Bladder emptying
1AH0.00	Incomplete emptying of bladder
1AZ..00	Genitourinary symptoms NOS
1AZ3.00	Difficulty with micturition
1AZ6.00	Lower urinary tract symptoms
1AZ6000	Mild lower urinary tract symptoms

1AZ6100	Moderate lower urinary tract symptoms
1AZ6200	Severe lower urinary tract symptoms
1AZZ.00	Genitourinary symptom NOS
1J4..00	Suspected UTI
46B..00	Urine bacteriuria test
46B3.00	Urine bacteria test: positive
46B4.00	Urinary pneumococcal antigen test
46BZ.00	Urine bacteria test NOS
46f..00	Urine leucocyte test
46f2.00	Urine leucocyte test = +
46f3.00	Urine leucocyte test = ++
46f4.00	Urine leucocyte test = +++
46f5.00	Urine leucocyte test = trace
46G..00	Urine microscopy: cells
46G4.11	Leucocytes in urine
46G4.12	Sterile pyuria
46G5.00	Urine micr.: leucs - % polys
46G8.00	Urine Microscopy: white cells
46GZ.00	Urine microscopy: cells NOS
K1...00	Other urinary system diseases
K10..00	Infections of kidney
K10..11	Renal infections
K100.00	Chronic pyelonephritis
K100000	Chronic pyelonephritis without medullary necrosis
K100100	Chronic pyelonephritis with medullary necrosis
K100200	Chronic pyelitis
K100300	Chronic pyonephrosis
K100400	Nonobstructive reflux-associated chronic pyelonephritis
K100500	Chronic obstructive pyelonephritis
K100600	Calculous pyelonephritis
K100z00	Chronic pyelonephritis NOS
K101.00	Acute pyelonephritis
K101000	Acute pyelonephritis without medullary necrosis
K101200	Acute pyelitis
K101300	Acute pyonephrosis
K101z00	Acute pyelonephritis NOS
K102.00	Renal and perinephric abscess
K102000	Renal abscess
K102100	Perinephric abscess
K102200	Renal carbuncle
K102z00	Renal and perinephric abscess NOS
K103.00	Pyeloureteritis cystica
K103.11	Ureteritis cystica
K103.12	Infestation of renal pelvis with ureter
K104.00	Xanthogranulomatous pyelonephritis

K105.00	Chronic infective interstitial nephritis
K106.00	Candida pyelonephritis
K10y.00	Pyelonephritis and pyonephrosis unspecified
K10y000	Pyelonephritis unspecified
K10y100	Pyelitis unspecified
K10y200	Pyonephrosis unspecified
K10y300	Pyelonephritis in diseases EC
K10y400	Pyelitis in diseases EC
K10yz00	Unspecified pyelonephritis NOS
K10z.00	Infection of kidney NOS
K112.00	Hydronephrosis with renal and ureteral calculous obstruction
K113.00	Hydronephrosis with ureteropelvic junction obstruction
K113.11	Hydronephrosis with pelviureteric junction obstruction
K11X.00	Hydronephrosis with ureteral stricture NEC
K11z.00	Hydronephrosis NOS
K12..00	Calculus of kidney and ureter
K12..11	Kidney calculus
K12..12	Urinary calculus
K120.00	Calculus of kidney
K120.11	Nephrolithiasis NOS
K120.12	Renal calculus
K120.13	Renal stone
K120000	Staghorn calculus
K120z00	Renal calculus NOS
K121.00	Calculus of ureter
K121.11	Ureteric calculus
K121.12	Ureteric stone
K121.13	Ureterolithiasis
K122.00	Calculus of kidney with calculus of ureter
K12z.00	Urinary calculus NOS
K13..00	Other kidney and ureter disorders
K13..11	Other kidney disorders
K13..12	Other ureter disorders
K132.00	Acquired cyst of kidney
K132.11	Acquired renal cystic disease
K132000	Single acquired kidney cyst
K132100	Multiple acquired kidney cysts
K132200	Peripelvic (lymphatic) cyst
K132300	Acquired renal cyst with neoplastic change
K132400	Acquired renal cyst without neoplastic change
K132z00	Acquired cyst of kidney NOS
K133.00	Stricture of ureter
K133000	Postoperative ureteric constriction
K133100	Stricture of pelviureteric junction

K133z00	Stricture of ureter NOS
K134.00	Other ureteric obstruction
K134z00	Occlusion of ureter NOS
K135.00	Hydroureter
K136.11	Orthostatic proteinuria
K137.00	Vesicoureteric reflux
K137.11	Ureteric reflux
K13B.00	Calyceal diverticulum
K13y600	Ureterocele - acquired
K13y611	Idiopathic dilation of ureter
K13y700	Megaloureter - acquired
K13y800	Perirenal haematoma
K13y900	Ureteric neuromuscular incoordination
K13yA00	Dent's disease
K13yz00	Other kidney and ureteric disorders NOS
K13z.00	Kidney and ureter disease NOS
K14..00	Lower urinary tract calculus
K140.00	Bladder calculus
K140.11	Bladder stone
K140000	Calculus in diverticulum of bladder
K140100	Other calculus in bladder
K140z00	Bladder calculus NOS
K141.00	Calculus in urethra
K14y.00	Other lower urinary tract calculus
K14z.00	Lower urinary tract calculus NOS
K15..00	Cystitis
K150.00	Acute cystitis
K151.00	Chronic interstitial cystitis
K151000	Hunner's ulcer
K151100	Panmural fibrosis of bladder
K151200	Submucous cystitis
K151z00	Chronic interstitial cystitis NOS
K152.00	Other chronic cystitis
K152000	Subacute cystitis
K152y00	Chronic cystitis unspecified
K152z00	Other chronic cystitis NOS
K153.00	Trigonitis
K153.11	Follicular cystitis
K153000	Acute trigonitis
K153100	Chronic trigonitis
K153200	Urethrotrigonitis
K153z00	Trigonitis NOS
K154.00	Cystitis in diseases EC
K154000	Cystitis in actinomycosis
K154100	Cystitis in amoebiasis
K154200	Cystitis in bilharziasis

K154500	Cystitis in gonorrhoea
K154600	Cystitis in moniliasis
K154700	Cystitis in trichomoniasis
K154800	Cystitis in tuberculosis
K154z00	Cystitis in diseases EC NOS
K155.00	Recurrent cystitis
K15y.00	Other specified cystitis
K15y000	Cystitis cystica
K15y100	Irradiation cystitis
K15y200	Abscess of bladder
K15y300	Malakoplakia of bladder
K15yz00	Other cystitis NOS
K15z.00	Cystitis NOS
K16..00	Other disorders of bladder
K160.00	Bladder neck obstruction
K160.11	Contracture of bladder neck
K160.12	Stenosis of bladder neck
K160.13	BOO - Bladder outflow obstruction
K161.00	Intestinovesical fistula
K161000	Enterovesical fistula
K161100	Vesicocolic fistula
K161111	Colovesical fistula
K161200	Vesicosigmoidal fistula
K161300	Vesicorectal fistula
K161z00	Intestinovesical fistula NOS
K162.00	Vesical fistula NEC
K162000	Vesicocutaneous fistula
K162100	Vesicoperineal fistula
K162200	Urethrovesical fistula
K162z00	Vesical fistula NEC NOS
K163.00	Diverticulum of bladder
K163000	Acquired bladder diverticulum
K163100	False bladder diverticulum
K163200	Bladder diverticulitis
K163z00	Diverticulum of bladder NOS
K164.00	Atony of bladder
K164.11	Atonic bladder
K164000	Hypotonic bladder
K164100	Bladder inertia
K164z00	Atony of bladder NOS
K165.00	Other functional disorder of bladder
K165000	Hypertonic bladder sphincter
K165100	Bladder sphincter paralysis
K165200	Bladder outflow obstruction
K165300	Detrusor instability
K165400	Unstable bladder

K165z00	Other bladder function disorder NOS
K166.00	Bladder rupture due to nontraumatic cause
K167.00	Haemorrhage into bladder wall
K168.00	Amyloid of bladder
K16V.00	Neuromuscular dysfunction of bladder, unspecified
K16V000	Neuropathic bladder
K16V011	Neurogenic bladder
K16V100	Overactive bladder
K16W.00	Reflex neuropathic bladder, not elsewhere classified
K16X.00	Uninhibited neuropathic bladder, NEC
K16y.00	Other bladder disorders
K16y000	Calcified bladder
K16y100	Contracted bladder
K16y200	Bladder haemorrhage
K16y300	Bladder hypertrophy
K16y400	Irritable bladder
K16y411	Detrusor instability
K16y412	Unstable bladder
K16y500	Trabeculation of bladder
K16y700	Squamous metaplasia of bladder
K16y800	Functional disorder of bladder
K16y811	Functional voiding disorder
K16y900	Metaplasia of trigone
K16yA00	Bladder scarring
K16yz00	Other bladder disorders NOS
K16z.00	Bladder disorders NOS
K17..00	Urethritis due to non venereal causes
K17..11	Periurethritis
K170.00	Urethral and periurethral abscess
K170.11	Urethral abscess
K170000	Urethral abscess unspecified
K170100	Bulbourethral gland abscess
K170111	Cowper's gland abscess
K170200	Urethral gland abscess
K170300	Periurethral cellulitis
K170311	Periurethritis
K170400	Periurethral abscess
K170z00	Urethral abscess NOS
K171.00	Post menopausal atrophic urethritis
K171.11	Post menopausal urethritis
K172.00	Candidal urethritis
K17y.00	Other urethritis
K17y000	Urethritis unspecified
K17y100	Urethral syndrome NOS
K17y200	Skene's glands adenitis

K17y300	Cowperitis
K17y400	Urethral meatitis
K17y500	Urethral meatal ulcer
K17y600	Verumontanitis
K17y700	Utriculus masculinus
K17yz00	Other urethritis NOS
K17z.00	Urethritis due to non venereal cause NOS
K18..00	Urethral stricture
K18..11	Pinhole meatus
K180.00	Infective urethral stricture
K180000	Urethral stricture due to unspecified infection
K180100	Urethral stricture due to infection EC
K180z00	Infective urethral stricture NOS
K181.00	Traumatic urethral stricture
K181.11	Postobstetric urethral stricture
K182.00	Postoperative urethral stricture
K182.11	Postcatheterisation urethral stricture
K183.00	Stenosis of urinary meatus
K18y.00	Other urethral stricture
K18z.00	Urethral stricture NOS
K19..00	Other urethral and urinary tract disorders
K19..11	Other urethral disorders
K190.00	Urinary tract infection, site not specified
K190.11	Recurrent urinary tract infection
K190000	Bacteriuria, site not specified
K190011	Asymptomatic bacteriuria
K190100	Pyuria, site not specified
K190200	Post operative urinary tract infection
K190300	Recurrent urinary tract infection
K190311	Recurrent UTI
K190400	Chronic urinary tract infection
K190500	Urinary tract infection
K190600	Urosepsis
K190X00	Persistent proteinuria, unspecified
K190z00	Urinary tract infection, site not specified NOS
K191.00	Urethral fistula
K191000	Urethroperineal fistula
K191100	Urethrorectal fistula
K191z00	Urethral fistula NOS
K192.00	Urethral diverticulum
K193.00	Urethral caruncle
K193.11	Urethral polyp
K194.00	Urethral false passage
K195.00	Prolapsed urethral mucosa
K195.11	Urethrocele
K196.00	Urinary obstruction unspecified

K196.11	Obstructive uropathy, unspecified
K197.00	Haematuria
K197.11	Traumatic haematuria
K197.12	Essential haematuria
K197000	Painless haematuria
K197100	Painful haematuria
K197200	Microscopic haematuria
K197300	Frank haematuria
K197400	Clot haematuria
K197500	Benign familial haematuria
K198.00	Stress incontinence
K19C.00	Other obstructive and reflux uropathy
K19W.00	Urethral disorder, unspecified
K19X.00	Obstructive and reflux uropathy, unspecified
K19y.00	Other urinary tract disorders
K19y000	Urethral rupture due to nontraumatic cause
K19y100	Urethral cyst
K19y200	Urethral granuloma
K19y300	Pneumaturia
K19y400	Bleeding from urethra
K19y411	Urethral bleeding
K19yw00	Disorder of urinary system, unspecified
K19yz00	Other urinary tract disorders NOS
K19z.00	Urethral and urinary tract disorders NOS
K1A..00	Urinary calculus in schistosomiasis
K1y..00	Other specified diseases of urinary system
K1z..00	Other urinary system diseases NOS
Ky...00	Other specified diseases of genitourinary system
Kyu..00	[X]Additional genitourinary disease classification terms
Kyu1200	[X]Other obstructive and reflux uropathy
Kyu1300	[X]Obstructive and reflux uropathy, unspecified
Kyu1F00	[X]Hydronephrosis with ureteral stricture NEC
Kyu3.00	[X]Urolithiasis
Kyu3000	[X]Other lower urinary tract calculus
Kyu3100	[X]Calculus of urinary tract in other diseases CE
Kyu4.00	[X]Other disorders of kidney and ureter
Kyu4100	[X]Other specified disorders of kidney and ureter
Kyu5.00	[X]Other diseases of urinary system
Kyu5000	[X]Other chronic cystitis
Kyu5100	[X]Other cystitis
Kyu5200	[X]Other neuromuscular dysfunction of bladder
Kyu5300	[X]Other specified disorders of bladder
Kyu5500	[X]Other urethritis
Kyu5600	[X]Other urethral stricture

Kyu5A00	[X]Other specified urinary incontinence
Kyu5B00	[X]Other specified disorders of urinary system
Kyu5E00	[X]Neuromuscular dysfunction of bladder, unspecified
Kyu5F00	[X]Urethral disorder, unspecified
Kyu6100	[X]Other specified disorders of prostate
Kyu8200	[X]Other diseases of Bartholin's gland
Kyu8800	[X]Disease of Bartholin's gland, unspecified
Kyu9200	[X]Other female urinary-genital tract fistulae
Kyu9300	[X]Other female intestinal-genital tract fistulae
Kyu9400	[X]Other female genital tract fistulae
Kyu9F00	[X]Other specified menopausal and perimenopausal disorders
KyuA.00	[X]Other disorders of genitourinary tract
KyuA000	[X]Other postprocedural disorders/genitourinary system
L162.00	Unspecified renal disease in pregnancy
L162.11	Albuminuria in pregnancy without hypertension
L162.12	Nephropathy NOS in pregnancy without hypertension
L162.13	Uraemia in pregnancy without hypertension
L162000	Unspecified renal disease in pregnancy unspecified
L162100	Unspecified renal disease in pregnancy - delivered
L165.00	Asymptomatic bacteriuria in pregnancy
L165200	Asymptomatic bacteriuria in pregnancy - del with p/n comp
L165300	Asymptomatic bacteriuria in pregnancy - not delivered
L165z00	Asymptomatic bacteriuria in pregnancy NOS
L166.00	Genitourinary tract infections in pregnancy
L166.11	Cystitis of pregnancy
L166000	Genitourinary tract infection in pregnancy unspecified
L166100	Genitourinary tract infection in pregnancy - delivered
L166300	Genitourinary tract infection in pregnancy - not delivered
L166400	Genitourinary tract infection in pregnancy with p/n comp
L166500	Infections of kidney in pregnancy
L166600	Urinary tract infection following delivery
L166700	Infections of the genital tract in pregnancy
L166800	Urinary tract infection complicating pregnancy
L166z00	Genitourinary tract infection in pregnancy NOS
L166z11	UTI - urinary tract infection in pregnancy
L16A.00	Glycosuria during pregnancy
L16A000	Glycosuria during pregnancy unspecified
L16A100	Glycosuria during pregnancy - delivered
L16A200	Glycosuria during pregnancy - delivered with p/n comp

L16A300	Glycosuria during pregnancy - not delivered
L16Az00	Glycosuria during pregnancy NOS
L177.00	Infections of bladder in pregnancy
L178.00	Infections of urethra in pregnancy
L1y..00	Complications of pregnancy/childbirth/puerperium OS
L1z..00	Complications of pregnancy/childbirth/puerperium NOS
L3z..00	Complications of labour and delivery NOS
PDz..00	Urinary system anomalies NOS
PDz0.00	Unspecified anomaly of kidney
PDz2.00	Unspecified anomaly of bladder
PDz3.00	Unspecified anomaly of urethra
SP07Q00	Catheter-associated urinary tract infection
SP07Q11	CAUTI - catheter-associated urinary tract infection

Supplementary Table 4: Read codes for skin conditions.

readcode	readterm
14F..00	H/O: skin disorder
14F3.00	H/O: chronic skin ulcer
14F4.00	H/O: Admission in last year for diabetes foot problem
14F5.00	H/O: venous leg ulcer
14F6.00	H/O: foot ulcer
14F7.00	H/O: arterial lower limb ulcer
14FZ.00	H/O: skin disease NOS
1D14.00	C/O: a rash
1N0..00	Skin symptoms
1N00.00	Change in skin lesion
1N04.00	Itching of skin lesion
1N05.00	Mottling of skin
2F7..00	O/E - pustules
2F72.00	O/E - pustules present
2F73.00	O/E - purulent pustules
2F74.00	O/E - deep seated pustules
2F75.00	O/E - follicular pustules
2F7Z.00	O/E - pustules NOS
2FD..00	O/E - skin cyst
2FD2.00	O/E - skin cyst present
2FD2000	O/E - eyebrow cyst present
2FD2100	O/E - scalp cyst present
2FDZ.00	O/E - skin cyst NOS
2FF..00	O/E - skin ulcer
2FF2.00	O/E - skin ulcer present
2FF3.00	O/E - depth of ulcer
2FFZ.00	O/E - skin ulcer NOS
2G2A.00	Tinel's sign
2G35.00	O/E - nails - pitting
2G37.00	Splitting toenail
2G48.00	O/E - ankle ulcer
2G51000	Foot abnormality - diabetes related
2G54.00	O/E - Right foot ulcer
2G55.00	O/E - Left foot ulcer
2G5A.00	O/E - Right diabetic foot at risk
2G5B.00	O/E - Left diabetic foot at risk
2G5C.00	Foot abnormality - diabetes related
2G5d.00	O/E - Left diabetic foot at increased risk
2G5E.00	O/E - Right diabetic foot at low risk
2G5e.00	O/E - Right diabetic foot at increased risk
2G5F.00	O/E - Right diabetic foot at moderate risk
2G5G.00	O/E - Right diabetic foot at high risk

2G5H.00	O/E - Right diabetic foot - ulcerated
2G5I.00	O/E - Left diabetic foot at low risk
2G5J.00	O/E - Left diabetic foot at moderate risk
2G5K.00	O/E - Left diabetic foot at high risk
2G5L.00	O/E - Left diabetic foot - ulcerated
2G5S.00	O/E - right healed foot ulcer
2G5V.00	O/E - right chronic diabetic foot ulcer
2G5W.00	O/E - left chronic diabetic foot ulcer
2G64.00	O/E - infected toe
A35..00	Erysiplas
A90..00	Congenital syphilis
A900.00	Early congenital syphilis with symptoms
A900.12	Congenital syphilitic choroiditis
A900.13	Congenital syphilitic chronic coryza
A900.14	Congenital syphilitic epiphysitis
A900.16	Congenital syphilitic osteochondritis
A901.00	Early latent congenital syphilis
A902.00	Early congenital syphilis NOS
A903.00	Syphilitic interstitial keratitis
A904.00	Juvenile neurosyphilis
A904200	Congenital syphilitic meningitis
A905.00	Other late congenital syphilis
A905000	Congenital syphilitic gumma
A905100	Hutchinson's teeth
A905200	Syphilitic saddle nose
A905300	Late congenital syphilitic oculopathy
A906.00	Latent late congenital syphilis
A907.00	Unspecified late congenital syphilis
A90z.00	Congenital syphilis NOS
A913.00	Secondary syphilis of skin or mucus membranes
A913000	Secondary syphilis of anus
A913300	Secondary syphilis of skin
A913400	Secondary syphilis of tonsils
A913500	Secondary syphilis of vulva
A913z00	Secondary syphilis of skin or mucus membranes NOS
A92..00	Latent early syphilis
A92z.00	Latent early syphilis NOS
M0...00	Skin and subcutaneous tissue infections
M00..00	Carbuncle
M000.00	Carbuncle of face
M000000	Carbuncle of ear
M000100	Carbuncle of face (excluding eye)
M000200	Carbuncle of nasal septum
M000300	Carbuncle of temple region
M000z00	Carbuncle of face NOS

M001.00	Carbuncle of neck
M002.00	Carbuncle of trunk
M002000	Carbuncle of chest wall
M002100	Carbuncle of breast
M002200	Carbuncle of back
M002300	Carbuncle of abdominal wall
M002400	Carbuncle of umbilicus
M002500	Carbuncle of flank
M002600	Carbuncle of groin
M002700	Carbuncle of perineum
M002z00	Carbuncle of trunk NOS
M003.00	Carbuncle of upper arm and forearm
M003000	Carbuncle of shoulder
M003100	Carbuncle of axilla
M003200	Carbuncle of upper arm
M003300	Carbuncle of elbow
M003400	Carbuncle of forearm
M003z00	Carbuncle of upper arm and forearm NOS
M004.00	Carbuncle of hand
M004000	Carbuncle of wrist
M004100	Carbuncle of thumb
M004200	Carbuncle of finger
M004z00	Carbuncle of hand NOS
M005.00	Carbuncle of buttock
M005000	Carbuncle of anus
M005100	Carbuncle of gluteal region
M005z00	Carbuncle of buttock NOS
M006.00	Carbuncle of leg (excluding foot)
M006000	Carbuncle of hip
M006100	Carbuncle of thigh
M006200	Carbuncle of knee
M006300	Carbuncle of lower leg
M006400	Carbuncle of ankle
M006z00	Carbuncle of leg (excluding foot) NOS
M007.00	Carbuncle of foot
M007100	Carbuncle of heel
M007200	Carbuncle of toe
M007z00	Carbuncle of foot NOS
M00y.00	Carbuncle of other specified site
M00y000	Carbuncle of head (excluding face)
M00yz00	Carbuncle of other specified site NOS
M00z.00	Carbuncle NOS
M01..00	Furuncle - boil
M010.00	Boil of face
M010000	Boil of ear
M010100	Boil of face (excluding eye)

M010200	Boil of nasal septum
M010300	Boil of temple region
M010400	Boil of external nose
M010z00	Boil of face NOS
M011.00	Boil of neck
M012.00	Boil of trunk
M012000	Boil of chest wall
M012100	Boil of breast
M012200	Boil of back
M012300	Boil of abdominal wall
M012400	Boil of umbilicus
M012500	Boil of flank
M012600	Boil of groin
M012700	Boil of perineum
M012z00	Boil of trunk NOS
M013.00	Boil of upper arm and forearm
M013000	Boil of shoulder
M013100	Boil of axilla
M013200	Boil of upper arm
M013300	Boil of elbow
M013400	Boil of forearm
M013z00	Boil of upper arm and forearm NOS
M014.00	Boil of hand
M014000	Boil of wrist
M014100	Boil of thumb
M014200	Boil of finger
M014z00	Boil of hand NOS
M015.00	Boil of buttock
M015000	Boil of anus
M015100	Boil of gluteal region
M015z00	Boil of buttock NOS
M016.00	Boil of leg (excluding foot)
M016000	Boil of hip
M016100	Boil of thigh
M016200	Boil of knee
M016300	Boil of lower leg
M016400	Boil of ankle
M016z00	Boil of leg (excluding foot) NOS
M017.00	Boil of foot
M017000	Boil of foot unspecified
M017100	Boil of heel
M017200	Boil of toe
M017z00	Boil of foot NOS
M01y.00	Boil of other specified site
M01y000	Boil of head (excluding face)
M01yz00	Boil of other specified site NOS

M01z.00	Boil NOS
M01z.11	Recurrent boils
M01z.12	Boils of multiple sites
M01z000	Multiple boils
M02..00	Cellulitis and abscess of finger and toe
M020.00	Cellulitis and abscess of finger
M020000	Cellulitis and abscess of finger unspecified
M020100	Finger pulp abscess
M020111	Felon
M020112	Whitlow
M020200	Onychia of finger
M020300	Paronychia of finger
M020311	Perionychia of finger
M020400	Finger web space infection
M020500	Pulp space infection of finger/thumb
M020z00	Cellulitis and abscess of finger NOS
M021.00	Cellulitis and abscess of toe
M021000	Cellulitis and abscess of toe unspecified
M021100	Onychia of toe
M021200	Paronychia of toe
M021300	Pulp space infection of toe
M021z00	Cellulitis and abscess of toe NOS
M021z11	Perionychia of toe
M02z.00	Cellulitis and abscess of digit NOS
M02z.11	Nail infection NOS
M02z.12	Paronychia
M02z.13	Infected nailfold
M02z.14	Nailfold infected
M03..00	Other cellulitis and abscess
M03..11	Abscess of skin area excluding digits of hand or foot
M03..12	Acute lymphangitis of skin excluding digits of hand or foot
M03..13	Cellulitis of skin area excluding digits of hand or foot
M030.00	Cellulitis and abscess of face
M030000	Cellulitis and abscess of cheek (external)
M030011	Cellulitis and abscess of cheek
M030100	Cellulitis and abscess of nose (external)
M030111	Cellulitis and abscess of nose
M030200	Cellulitis and abscess of chin
M030300	Cellulitis and abscess of submandibular region
M030400	Cellulitis and abscess of forehead
M030500	Cellulitis and abscess of temple region
M030600	Cellulitis of face
M030z00	Cellulitis and abscess of face NOS
M031.00	Cellulitis and abscess of neck

M032.00	Cellulitis and abscess of trunk
M032000	Cellulitis and abscess of chest wall
M032100	Cellulitis and abscess of breast
M032200	Cellulitis and abscess of back
M032300	Cellulitis and abscess of abdominal wall
M032400	Cellulitis and abscess of umbilicus
M032500	Cellulitis and abscess of flank
M032600	Cellulitis and abscess of groin
M032700	Cellulitis and abscess of perineum
M032800	Cellulitis of trunk
M032z00	Cellulitis and abscess of trunk NOS
M033.00	Cellulitis and abscess of arm
M033000	Cellulitis and abscess of shoulder
M033100	Cellulitis and abscess of axilla
M033200	Cellulitis and abscess of upper arm
M033300	Cellulitis and abscess of elbow
M033400	Cellulitis and abscess of forearm
M033z00	Cellulitis and abscess of arm NOS
M034.00	Cellulitis and abscess of hand excluding digits
M034.11	Cellulitis and abscess of hand
M034000	Cellulitis and abscess of hand unspecified
M034011	Abscess of dorsum of hand
M034012	Abscess of palm of hand
M034013	Cellulitis of dorsum of hand
M034014	Cellulitis of palm of hand
M034100	Cellulitis and abscess of wrist
M034z00	Cellulitis and abscess of hand NOS
M035.00	Cellulitis and abscess of buttock
M036.00	Cellulitis and abscess of leg excluding foot
M036.11	Cellulitis and abscess of leg
M036000	Cellulitis and abscess of hip
M036100	Cellulitis and abscess of thigh
M036200	Cellulitis and abscess of knee
M036300	Cellulitis and abscess of lower leg
M036400	Cellulitis and abscess of ankle
M036z00	Cellulitis and abscess of leg NOS
M037.00	Cellulitis and abscess of foot excluding toe
M037.11	Cellulitis and abscess of foot
M037000	Cellulitis and abscess of foot unspecified
M037100	Cellulitis and abscess of heel
M037200	Cellulitis in diabetic foot
M037z00	Cellulitis and abscess of foot NOS
M038.00	Cellulitis of external ear
M03y.00	Other specified cellulitis and abscess
M03y000	Cellulitis and abscess of head unspecified
M03y011	Abscess of scalp

M03z.00	Cellulitis and abscess NOS
M03z000	Cellulitis NOS
M03z100	Abscess NOS
M03zz00	Cellulitis and abscess NOS
M03zz11	Acute lymphangitis NOS
M04..00	Acute lymphadenitis
M04..11	Acute abscess lymph node
M04..12	Acute adenitis
M040.00	Acute lymphadenitis of trunk
M041.00	Acute lymphadenitis of upper limb
M042.00	Acute lymphadenitis of lower limb
M043.00	Acute lymphadenitis of face, head and neck
M05..00	Impetigo
M050.00	Impetigo contagiosa unspecified
M051.00	Impetigo contagiosa bullosa
M052.00	Impetigo contagiosa gyrata
M053.00	Impetigo circinata
M054.00	Impetigo neonatorum
M055.00	Impetigo simplex
M056.00	Impetigo follicularis
M057.00	Chronic symmetrical impetigo
M05z.00	Impetigo NOS
M06..00	Pilonidal sinus/cyst
M060.00	Pilonidal cyst with abscess
M061.00	Pilonidal cyst with no abscess
M061.11	Dermal sinus
M062.00	Pilonidal sinus with abscess
M063.00	Pilonidal sinus without abscess
M06z.00	Pilonidal sinus/cyst NOS
M07..00	Other local infections of skin and subcutaneous tissue
M070.00	Pyoderma
M070.11	Purulent dermatitis
M070000	Pyoderma chancriforme
M070100	Pyoderma faciale
M070200	Pyoderma gangrenosum
M070300	Pyoderma ulcerosum tropicalum
M070z00	Pyoderma NOS
M071.00	Pyogenic granuloma
M071000	Pyogenic granuloma unspecified
M071100	Pyogenic progressive granuloma
M071200	Granuloma telangiectaticum
M071300	Umbilical granuloma
M071z00	Pyogenic granuloma NOS
M072.00	Erythrasma
M073.00	Scalp infection

M07y.00	Local infection of skin or subcutaneous tissue OS
M07y.11	Pustular eczema
M07y000	Pustular bacterid
M07y100	Ecthyma
M07y200	Dermatitis vegetans
M07y300	Perleche
M07y400	Pitted keratolysis
M07y500	Inflammation of scar
M07yz00	Other spec local skin/subc infection NOS
M07yz11	Infection toe
M07yz12	Infection foot
M07yz13	Infection finger
M07z.00	Local infection skin/subcut tissue NOS
M07z.11	Infected insect bite
M07z.12	Infected skin ulcer
M07z.13	Septic spots
M07z.14	Infected dermatitis
M07z.15	Sinus
M07z000	Infection foot
M07z100	Infection toe
M07z200	Infection finger
M08..00	Cutaneous cellulitis
M080.00	[X]Cellulitis of finger and toe
M080.11	[X]Nail bed infection
M080.12	[X]Septic thumb
M080.13	[X]Cellulitis of thumb
M081.00	[X]Cellulitis of other parts of limb
M082.00	Cellulitis of face
M083.00	Cellulitis of trunk
M084.00	[X]Cellulitis of breast
M085.00	Cellulitis of leg
M086.00	Cellulitis of ankle
M087.00	Chronic paronychia
M088.00	Cellulitis of arm
M089.00	Cellulitis of neck
M08A.00	Cellulitis of axilla
M08B.00	Cellulitis of foot
M08C.00	Cellulitis of toe
M08y.00	[X]Cellulitis of other sites
M09..00	Cutaneous abscess
M090.00	[X]Abscess of face
M091.00	[X]Abscess of neck
M092.00	[X]Abscess of trunk
M092000	[X]Abscess of buttock
M092100	[X]Abdominal wall abscess

M092200	[X]Perineal abscess
M093.00	[X]Abscess of buttock
M094.00	[X]Abscess of limb
M094000	[X]Abscess of axilla
M095.00	Skin abscess
M09y.00	[X]Abscess of other site
M0y..00	Other specified infections of skin or subcutaneous tissue
M0z..00	Skin and subcut tissue infection NOS
M0z..11	Infected sebaceous cyst
M111.00	Atopic dermatitis/eczema
M153.00	Rosacea
M153000	Acne rosacea
M153100	Rhinophyma
M153200	Rosacea hypertrophica
M153300	Lupoid rosacea
M153400	Ocular rosacea
M153500	Perioral dermatitis
M153511	Circumoral dermatitis
M153600	Periocular dermatitis
M153z00	Rosacea NOS
M244.00	Folliculitis
M25y100	Hidradenitis
M25y111	Hidradenitis suppurativa
M25y600	Acne keloid
M25yX00	Apocrine sweat disorder, unspecified
M26..00	Sebaceous gland diseases
M260.00	Acne varioliformis
M260000	Acne frontalis
M260z00	Acne varioliformis NOS
M260z11	Acne necrotica
M261.00	Other acne
M261000	Acne vulgaris
M261011	Blackhead
M261012	Comedo
M261100	Acne conglobata
M261200	Bromine acne
M261300	Chlorine acne
M261400	Iodine acne
M261500	Colloid acne
M261600	Cystic acne
M261700	Acne neonatorum
M261800	Infantile acne
M261900	Occupational acne
M261A00	Pustular acne
M261B00	Steroid acne

M261C00	Tropical acne
M261D00	Acne urticata
M261E00	Acne excoriee des jeunes filles
M261F00	Acne fulminans
M261G00	Acne agminata
M261H00	Acne keloid
M261J00	Acne necrotica
M261K00	Acne keloidalis
M261X00	Acne, unspecified
M261z00	Other acne NOS
M262.00	Sebaceous cyst - wen
M262.11	Keratin cyst
M262.12	Sebaceous cyst
M262000	Trichilemmal cyst
M262100	Pilar cyst
M262200	Pilar cyst of scalp
M262211	Sebaceous cyst of scalp
M263.00	Seborrhoea
M263000	Seborrhoea corporis
M263100	Seborrhoea faciei
M263200	Seborrhoea nasi
M263300	Seborrhoea oleosa
M263400	Post-encephalitic seborrhoea
M263z00	Seborrhoea NOS
M26y.00	Other specified sebaceous gland diseases
M26y000	Asteatosis cutis
M26y200	Giant comedo
M26y300	Fordyce spots
M26y400	Sebaceous gland hypertrophy
M26yz00	Other sebaceous gland diseases NOS
M26z.00	Sebaceous gland diseases NOS
M2yz.11	Skin lesion
Myu6800	[X]Other acne
R02..00	[D]Symptoms affecting skin and other integumentary tissue
R021.00	[D]Rash and other nonspecific skin eruption
R021000	[D]Exanthem
R021100	[D]Rash on genitals
R021z00	[D]Rash and other nonspecific skin eruption NOS
R021z11	[D]Spots
R022.00	[D]Local superficial swelling, mass or lump
R022000	[D]Swelling, local and superficial
R022100	[D]Mass, localized and superficial
R022200	[D]Lump, localized and superficial
R022300	[D]Nodule, subcutaneous

R022400	[D]Localized swelling, mass and lump, upper limb
R022500	[D]Localized swelling, mass and lump, lower limb
R022600	[D]Localized swelling, mass and lump, multiple sites
R022700	[D]Axillary lump
R022800	[D]Lump on back
R022900	[D]Foot lump
R022A00	[D]Shoulder lump
R022B00	[D]Lump on hand
R022C00	[D]Lump on knee
R022D00	[D]Lump on leg
R022E00	[D]Lump on shin
R022F00	[D]Lump on thigh
R022G00	[D]Finger lump
R022H00	[D]Wrist lump
R022I00	[D]Toe lump
R022J00	[D]Subungual swelling
R022K00	[D]Buttock swelling
R022z00	[D]Local superficial swelling, mass or lump NOS
R02z.00	[D]Skin symptoms NOS

Supplementary Table 5: Read codes for eye conditions.

readcode	readterm
1486	H/O: iritis
148Z.00	H/O: eye disorder NOS
F400.00	Purulent endophthalmitis
F400100	Acute endophthalmitis
F400200	Panophthalmitis
F400300	Chronic endophthalmitis
F400400	Vitreous abscess
F400500	Eye infection
F400z00	Purulent endophthalmitis NOS
F44..00	Disorders of iris and ciliary body
F44..11	Ciliary body disorders
F44..12	Iridocyclitis
F440.00	Acute and subacute iridocyclitis
F440.11	Iritis - acute
F440000	Unspecified acute iridocyclitis
F440100	Unspecified subacute iridocyclitis
F440200	Primary iridocyclitis
F440300	Recurrent iridocyclitis
F440400	Secondary infected iridocyclitis
F440600	Hypopyon
F440700	Diabetic iritis
F440z00	Acute or subacute iritis NOS
F441.00	Chronic iridocyclitis
F441.11	Chronic iritis
F441000	Unspecified chronic iridocyclitis
F441100	Chronic iridocyclitis due to disease EC
F441200	Chronic anterior uveitis
F441z00	Chronic iridocyclitis NOS
F442.00	Certain types of iridocyclitis
F442000	Fuchs' heterochromic cyclitis
F442200	Lens-induced iridocyclitis
F442300	Vogt-Koyanagi syndrome
F442z00	Certain types of cyclitis NOS
F443.00	Unspecified iridocyclitis
F443.11	Uveitis NOS
F443000	Anterior uveitis
F443100	Iritis
F444.00	Iris and ciliary body vascular disorders
F444z00	Iris and ciliary body vascular disorders NOS
F446.11	Uveal cysts
F446000	Idiopathic cyst of iris, ciliary body or anterior chamber
F44y.00	Other iris and ciliary body disorders

F44yz00	Other iris or ciliary body disorder NOS
F44z.00	Iris or ciliary body disorder NOS
F48..00	Visual disturbances
F481.00	Subjective visual disturbances
F481000	Unspecified subjective visual disturbance
F481100	Sudden visual loss
F481400	Other transient visual loss
F481700	Photophobia
F481800	Other visual discomfort
F481C00	Photopsia
F481D00	Visual halos
F481E00	Refractive diplopia
F481F00	Refractive polyopia
F481G00	Other visual distortion
F481K00	Visual hallucinations
F482.00	Diplopia (double vision)
F483.00	Other binocular vision disorders
F483000	Unspecified binocular vision disorder
F484.00	Visual field defects
F484000	Unspecified visual field defect
F484z00	Visual field defects NOS
F485.00	Colour vision deficiency
F48y.00	Other specified visual disturbance
F48y000	Blurred vision NOS
F48y011	Cloudy vision NOS
F48y012	Dull vision NOS
F48yz00	Other specified visual disturbance NOS
F48z.00	Visual disturbance NOS
F4A..00	Keratitis
F4A..11	Keratoconjunctivitis
F4A0.00	Corneal ulcer
F4A0.11	Dendritic ulcer
F4A0000	Unspecified corneal ulcer
F4A0100	Marginal corneal ulcer
F4A0200	Ring corneal ulcer
F4A0300	Central corneal ulcer
F4A0400	Hypopyon ulcer
F4A0411	Serpiginous ulcer
F4A0600	Perforated corneal ulcer
F4A0700	Mooren's ulcer
F4A0z00	Corneal ulcer NOS
F4A1.00	Dendritic keratitis
F4A2.00	Other superficial keratitis without conjunctivitis
F4A2000	Unspecified superficial keratitis
F4A2100	Punctate keratitis
F4A2111	Keratitic precipitates

F4A2112	Thygeson's superficial punctate keratitis
F4A2200	Nummular keratitis
F4A2300	Striate keratitis
F4A2400	Macular keratitis NOS
F4A2500	Filamentary keratitis
F4A2711	Arc-welders' keratitis
F4A2800	Photokeratitis NOS
F4A2z00	Other superficial keratitis without conjunctivitis NOS
F4A3.00	Specific keratoconjunctivitis
F4A3000	Phlyctenular keratoconjunctivitis
F4A3100	Vernal conjunctivitis of limbus and cornea
F4A3200	Keratoconjunctivitis sicca (excluding Sjogren's syndrome)
F4A3300	Exposure keratoconjunctivitis
F4A3400	Neurotrophic keratoconjunctivitis
F4A3z00	Specific keratoconjunctivitis NOS
F4A4.00	Other keratoconjunctivitis
F4A4000	Unspecified keratoconjunctivitis
F4A4100	Keratitis or keratoconjunctivitis in other exanthemata
F4A4z00	Other keratoconjunctivitis NOS
F4A5.00	Interstitial and deep keratitis
F4A5000	Unspecified interstitial keratitis
F4A5100	Diffuse interstitial keratitis
F4A5300	Corneal abscess
F4A5400	Keratitis due to syphilis
F4A5500	Keratitis due to tuberculosis
F4A5z00	Interstitial and deep keratitis NOS
F4Ay.00	Other forms of keratitis
F4Az.00	Keratitis NOS
F4B..00	Corneal opacity and other disorders of cornea
F4B..11	Corneal disorders
F4B0.00	Corneal scars and opacities
F4B0z00	Corneal scar or opacity NOS
F4B1.00	Corneal pigmentations and deposits
F4B1z00	Corneal pigmentation or deposit NOS
F4B2.00	Corneal oedema
F4B2z00	Corneal oedema NOS
F4B3.00	Corneal membrane changes
F4B3z00	Corneal membrane changes NOS
F4B4.00	Corneal degenerations
F4B4z00	Corneal degenerations NOS
F4B5000	Corneal dystrophy unspecified
F4B7100	Corneal ectasia
F4B7300	Corneal staphyloma
F4By000	Corneal hypoaesthesia

F4By100	Corneal anaesthesia
F4Bz.00	Corneal disorder NOS
F4C0.00	Acute conjunctivitis
F4C0.11	Eye infection
F4C0.12	Conjunctivitis
F4C0000	Unspecified acute conjunctivitis
F4C0011	Conjunctivitis
F4C0100	Serous conjunctivitis
F4C0200	Acute follicular conjunctivitis
F4C0300	Acute mucopurulent conjunctivitis
F4C0311	Sticky eye
F4C0400	Catarrhal conjunctivitis
F4C0500	Pseudomembranous conjunctivitis
F4C0511	Membranous conjunctivitis
F4C0600	Acute atopic conjunctivitis
F4C0611	Acute allergic conjunctivitis
F4C0z00	Acute conjunctivitis NOS
F4C1.00	Chronic conjunctivitis
F4C1000	Unspecified chronic conjunctivitis
F4C1100	Simple chronic conjunctivitis
F4C1200	Chronic follicular conjunctivitis
F4C1300	Vernal conjunctivitis
F4C1z00	Chronic conjunctivitis NOS
F4C2.00	Blepharoconjunctivitis
F4C2000	Unspecified blepharoconjunctivitis
F4C2100	Angular blepharoconjunctivitis
F4C2200	Contact blepharoconjunctivitis
F4C2z00	Blepharoconjunctivitis NOS
F4C3.00	Other and unspecified conjunctivitis
F4C3000	Unspecified conjunctivitis
F4C3100	Rosacea conjunctivitis
F4C3200	Conjunctivitis with mucocutaneous disorder
F4C3300	Bacterial conjunctivitis
F4C3z00	Other conjunctivitis NOS
F4Cy.00	Other conjunctival disorders
F4Cy000	Filarial infection of conjunctiva
F4Cy100	Ocular pemphigoid
F4D..00	Inflammation of eyelids
F4D0.00	Blepharitis
F4D0.11	Cellulitis of eyelids
F4D0000	Unspecified blepharitis
F4D0100	Ulcerative blepharitis
F4D0200	Squamous blepharitis
F4D0z00	Blepharitis NOS
F4D1.00	Hordeolum and other deep inflammation of eyelid

F4D1000	Hordeolum externum (stye)
F4D1100	Hordeolum internum (infected meibomian cyst)
F4D1111	Meibomian cyst infected
F4D1200	Abscess of eyelid
F4D1211	Boil of eyelid
F4D1212	Furuncle of eyelid
F4D1300	Meibomianitis
F4D1400	Cellulitis of eyelid
F4D1z00	Hordeolum and other deep inflammation of eyelid NOS
F4D2.00	Chalazion (meibomian cyst)
F4D3000	Eczematous eyelid dermatitis
F4D3100	Contact or allergic eyelid dermatitis
F4D3111	Allergic dermatitis - eyelid
F4D3112	Contact eczema - eyelids
F4D4.00	Infective eyelid dermatitis of types resulting in deformity
F4D5.00	Other eyelid infective dermatitis
F4D6.00	Parasitic eyelid infestation
F4Dy.00	Other eyelid inflammation
F4Dy000	Ulcer of eyelid
F4Dz.00	Eyelid inflammation NOS
F4F0.00	Dacryoadenitis
F4F0000	Unspecified dacryoadenitis
F4F0100	Acute dacryoadenitis
F4F0200	Chronic dacryoadenitis
F4F0z00	Dacryoadenitis NOS
F4F3.00	Acute and unspecified inflammation of lacrimal passages
F4F3.11	Dacryocystitis - acute
F4F3000	Unspecified dacryocystitis
F4F3100	Acute lacrimal canaliculitis
F4F3200	Acute dacryocystitis
F4F3300	Phlegmonous dacryocystitis
F4F3z00	Dacryocystitis NOS
F4F6000	Lacrimal fistula
F4G0.00	Acute inflammation of orbit
F4G0000	Unspecified acute orbit inflammation
F4G0100	Orbital cellulitis
F4G0200	Orbital abscess
F4G0300	Orbital periostitis
F4G0400	Orbital osteomyelitis
F4G0500	Tenonitis
F4G0z00	Acute inflammation of orbit NOS
F4G1.00	Chronic inflammation of orbit
F4G1000	Unspecified chronic inflammation of orbit
F4G1300	Parasitic infestation of orbit

F4G1z00	Chronic inflammation of orbit NOS
F4K0.00	Scleritis and episcleritis
F4K0.11	Episcleritis
F4K0.12	Scleritis
F4K0000	Unspecified scleritis
F4K0100	Episcleritis periodica fugax
F4K0200	Episcleritis periodica fugax
F4K0300	Anterior scleritis
F4K0400	Scleromalacia perforans
F4K0500	Sclerokeratitis
F4K0600	Brawny scleritis
F4K0700	Posterior scleritis
F4K0711	Sclerotenonitis
F4K0800	Scleral abscess
F4K0z00	Scleritis or episcleritis NOS
F4Kz.00	Eye and adnexa disorder NOS
F4Kz000	Unspecified disorder of eye
F4Kz100	Eye pain NOS
F4Kz200	Swelling of eye NOS
F4Kz300	Mass of eye NOS
F4Kz400	Redness of eye NOS
F4Kz411	Red eye NOS
F4Kz500	Discharge of eye NOS
F4Kzz00	Ill-defined eye disorder NOS
SD81000	Corneal abrasion
SG00.00	Corneal foreign body
SH0..12	Corneal burns
